# Hyperbaric oxygen exposure alleviate metabolic side-effects of olanzapine treatment and is associated with Langerhans islet proliferation in rats

**DOI:** 10.3389/pore.2022.1610752

**Published:** 2022-12-16

**Authors:** Mohammad AlQudah, Mohammad Khalifeh, Rasha Al-Azaizeh, Amr Masaadeh, Omar M. Al-Rusan, Husam K. Haddad

**Affiliations:** ^1^ Department of Pathology and Microbiology, Faculty of Medicine, Jordan University of Science and Technology, Irbid, Jordan; ^2^ Department of Veterinary Basic Sciences, Jordan University of Science and Technology, Irbid, Jordan; ^3^ University of Iowa Hospitals and Clinics, Iowa City, IA, United States; ^4^ Department of Pathology and Laboratory Medicine, School of Medicine, Emory University, Atlanta, GA, United States; ^5^ Department of Pathology and Laboratory Medicine, Ministry of Health, Amman, Jordan

**Keywords:** olanzapine, hyperbaric oxygen therapy, pancreatic Langerhans cells, metabolic disorders, insulin

## Abstract

**Introduction:** Olanzapine (OLZ) is one of the second-generation antipsychotics drugs (APDs) used to treat several psychiatric illnesses. Olanzapine treatment is often associated with many metabolic side effects in a dose dependent manner such as obesity, dyslipidemia and insulin resistance, induction of type II diabetes and acute pancreatitis in some patients.

**Methods:** Hyperbaric Oxygen therapy (HBOT) was investigated as a tool to mitigate olanzapine metabolic side effects in rats. Thirty-six female Sprague Dawley (SD) rats were divided into 4 groups; rats on olanzapine treatment either exposed to hyperbaric oxygen therapy (HBOOLZ) or left without exposure (OLZ) then non-treated rats that either exposed to hyperbaric oxygen therapy or left without exposure (control). Rats received Hyperbaric Oxygen therapy for 35 days at 2.4 atmospheres absolute (ATA) for 2.5 h daily followed by intraperitoneal injection of olanzapine at 10 mg/kg or placebo.

**Results:** Rats on either hyperbaric oxygen therapy or olanzapine had a significant loss in body weight. Olanzapine treatment showed a decrease in serum insulin level, triglyceride, highdensity lipoprotein (HDL) cholesterol, and lipase level but an increase in fasting blood sugar (FBS), insulin resistance index (HOMA-IR) and amylase, while rats’ exposure to hyperbaric oxygen therapy reversed these effects. The Pancreatic Langerhans islets were up-regulated in both hyperbaric oxygen therapy and olanzapine treatments but the combination (HBOOLZ) doubled these islets number.

**Discussion:** This study advocated that hyperbaric oxygen therapy can be an alternative approach to control or reverse many metabolic disorders (MDs) associatedwith olanzapine treatment. In addition, it seems that hyperbaric oxygen therapy positively affect the pancreatic Langerhans cells activity and architecture.

## Introduction

Olanzapine (OLZ), a thienobenzodiazepine derivative, it which has binding affinity for a broad range of neurotransmitter receptors. Olanzapine is a second generation of atypical antipsychotic agents (APDs) that is effective in the treatment of many disorders such as Schizophrenia, bipolar disorders whether it is mixed or manic episodes, anxiety, chemotherapy-induced nausea, sleep disorders and treatment-resistant depression [1, 2]. However, it has a high metabolic side effects, specifically when it is prescribed for long-term treatment or administrated at high doses [3, 4]. These side effects include weight gain, insulin resistance and hyperglycemia, new-onset diabetes, and diabetic ketoacidosis [5]. Moreover, olanzapine treatment is associated with altered meal patterns as well as increase the risk of dyslipidemia, decrease in fat oxidation that is secondary or pre-dispose factor to olanzapine-induced weight gain in patients [6–9].

The mechanism of antipsychotic-induced weight gain remains unclear as there are conflicting results in the literature about inducing weight gain after APDs treatment. It has been showed in animal models that the APDs cause hyperphagia followed by hyperglycemia [10], while a study reports in rats on olanzapine showed that diabetes did occur but without any weight gain [11]. Olanzapine appears to induce an increase in central body fat deposition, insulin, and triglyceride levels, suggesting that it is possible to induce insulin resistance development in rats [12]. It is generally noticed that the OLZ effect on weight gain and triglycerides serum concentration occur in a dose dependent manner as high doses treatment with OLZ causes inability to move and reach food, which thus consequently prevents weight gain compared to injections of low concentration doses [13, 14]. This effect occurs only when the drug is delivered to the animals by injection with high dose while oral administration with any doses usually results in increase in weight gain [15, 16]. In the contrary, OLZ induces insulin resistance regardless of the dose or route of administration and leads to impaired glucose tolerance especially in young population as well as has a primary risk factor for metabolic syndrome [12, 17–20]. Therefore, discontinuation of APDs has been observed and sometimes recommended to decrease the plasma glucose level [12, 21–24]. In addition, it was also documented that metabolic changes associated with OLZ treatment causes changes in pancreatic enzymes such as amylase and lipase [25, 26]. In addition, dyslipidemia and insulin resistance could be the earliest detectable metabolic abnormalities in patients treated with OLZ, which could eventually proceed to prediabetes, pancreatic β-cell failure and changes in the architecture of pancreas [11, 18, 19].

Physicians usually prescribe glucose lowering drugs such as metformin and rosiglitazone to mitigate the metabolic side effects of olanzapine therapy. Such practice is associated with some undesirable side effects. It was reported that metformin is only partially effective to reverse olanzapine-induced hyperglycemia [27] as well as results in some psychotic side effects [28]. Rosiglitazone worsen olanzapine-induced perturbations in glucose metabolism [29], thus may lead to worsening of coronary heart disease and increased risk of heart attack [30]. As an alternative approach, patients on olanzapine therapy were recommended to perform exhaustive exercise to protect against olanzapine-induced hyperglycemia and other metabolic side-effects associated with OLZ therapy [31]. However, muscle weakness and tardive dyskinesia which occur during or after treatment with olanzapine may cause difficulty in performing exercises [14, 32, 33]. Therefore, the need for unconventional approach to reduce the metabolic side effects of olanzapine that take into consideration the nature of schizophrenic clinical demonstration and side effect associated with OLZ treatment to these patients.

Due to its safe and non-invasive nature, hyperbaric oxygen treatment (HBOT) has emerged since the 1990s as a potential alternative treatment strategy for many cases such as diabetic foot ulcers, osteomyelitis, and certain infections. Based on data obtained in many studies, giving oxygen through hyperbaric therapy helps in cases of diabetes and ultimately improve a person’s quality of life through decrease in blood glucose levels and reduce insulin resistance. Oxygen is involved in cellular respiration which provides cells with their required energy to perform functions [34], in addition to its ability to improve metabolic changes [35]. Clinical studies have previously revealed the improvement in the control of hyperglycemia in patients with diabetes undergoing HBOT as well as preserved islet β-cell mass by stimulating proliferation, inhibiting apoptosis and suppressing insulitis [36]. Therefore, HBOT improves pancreatic cell function especially β-cell [37]. It also causes increment in insulin sensitivity in pancreatic tissues through its direct upregulation effect on oxidative phosphorylation process in pancreatic β-cell mitochondria that lead to upregulation in adenosine triphosphate (ATP) production. HBOT has also been reported to prevent hyperglycemia and improve muscle oxidative capacity in rodents with Type 2 diabetes [38, 39].

This study investigated the effect of HBO therapy on metabolic side effects associated with olanzapine therapy in rat model. The olanzapine-induced pathological changes in pancreatic cells also assessed in the presence and absence of HBOT. To the best of our knowledge, this is the first study that employs HBOT to control metabolic side effects associated with OLZ treatment. This approach is to replace the increase of physical activities recommendation given to patients to control side effects associated with OLZ treatment but with less burden on these patients which are usually unwilling to perform these activities due to their nature of psychotic behavior and the decrease locomotor ability following APDs treatment.

## Methods and materials

### Animals

Thirty-six female Sprague Dawley (SD) rats at age between 6 and 7 weeks, average weight around 140 g ± sd. The study design was approved by the Animal Care and Use Committee of Jordan University of Science and Technology which follows the animal care and uses guidelines (ILAR) [40]. All animals had free access to water and were individually caged, maintained on 12 h light–dark cycle (lights on at 07:00 h) and at a temperature of 19–22°C and 30%–40% humidity. The animals were randomly divided into 4 major groups; experimental groups received OLZ treatment and either exposed to high oxygen under pressure in the HBO chamber (i.e., HBOOLZ) (10 rats) or left without HBOT (i.e., OLZ) (10 rats). Control groups received placebo (i.e., diluent) but were either exposed to HBOT (8 rats) or left without it (i.e., Control) (8 rats).

#### Olanzapine treatment

Olanzapine solution was prepared by dissolving pure/raw compound of the drug (Gift from ALHikma, Jordan) in 0.1 N HCL in distilled water. The PH was adjusted at 5.5 PH with 0.01 N NaOH and the PBS was then added to reach the required final concentration (1 mg/ml). Rats received single dose of treatment intraperitoneally at 10 mg/kg daily for 35 days [41].

#### Oxygen therapy (HBOT) conditions

The HBOT chamber was flushed with pure oxygen from oxygen generator and then the chamber gets pressurized at a rate of 0.3 MPa (3 ATA) per 20 min. The therapy was performed on rats for 2 hours after the oxygen concentration in the chamber reached at 90%–95% with pressure inside chamber. Rats daily received HBOT between 8:00 AM and 11:00 AM o’clock before OLZ treatment for a period of 35 days.

#### Animal weight and samples collection

The rats were weighed weekly before entering the HBOT. The cumulative percentage of weight gain was calculated by the difference of weekly weight gain in comparison to the starting weight of each animal divided by the starting weight. Rats at the end of the experiment were fasted for 12 h before euthanization which was done by head decapitation using guillotine. Blood samples were collected after animals’ euthanization in plane tubes. Serum was then separated by centrifugation at 10,000x g 4°C for 15 min. Serum samples were stored at −20°C until analysis. Liver and pancreas were collected from these animals and directly placed in 10% neutral buffered formaldehyde in water.

#### Determination of the serum lipid profile and glucose concentrations

Serum total cholesterol (CHOL), low-density lipoprotein (LDL) cholesterol and triglycerides (TG) was performed according to manufacturer recommendation (Code number: 11579, 11805, 11528, respectively, BioSystems, Spain). The HDL-cholesterol (HDL-C) was analyzed using AGAPPE kit (Code number 52013001). TG, LDL and CHOL levels were evaluated in similar procedures provided by the manufacturer (Code number: 11579, 11805, 11528, respectively, BioSystems, Spain). The absorbance (A) of samples and standard was measured at 500 nm against reagent blank using spectrophotometer (UV/VIS spectrometer T80+, United States). The cholesterol concentration in the sample was calculated using the formula provided in the kits. For HDL-C measurements the absorbance of the reaction was measured at 505 nm. Serum glucose values were obtained after sampling using an enzymatic photometric test and were done in accordance of manufacturer recommendations (Code number: CH0280, ARCOMEX, Jordan). The absorbance of samples and standard was measured at 500 nm.

#### Enzymology

Pancreatic enzymes (i.e., amylase, lipase) and Liver enzymes (ALT and AST) were determined according to manufacturer recommendations. Amylase, ALT and AST were analyzed using Spinreact (Code number: 41202, 1001172, 1001162, respectively, Spinreact kit, Spain) while Lipase was detected using Biochemical Enterprise kit (Code number: LIP3542, Biochemical Enterprise, Italy).

#### Insulin and indicators of insulin resistance or sensitivity indices

Serum insulin was determined using a commercially available ELISA kit and following manufacturer recommendations (Code number: CEA448Ra, Cloud-Clone Corp, United States). All reagents and samples were brought to room temperature (18–25°C) before used. The color changes in microtiter plates were measured by ELISA reader (Biotek, United States) at 450 nm.

The following formulas was used to calculate Homeostasis model assessment of insulin resistance, the HOMA-IR index: insulin (mU/mL) X glucose (mmol/L)/22.5; and HOMA beta-cell function of the pancreas assessment, the HOMA β-cell index: (20 X fasting serum insulin (mU/mL))/(FBS (mmol/L)–3.50).

### Histological evaluation

The fixed pancreas specimens (10% formaldehyde) were dehydrated in a series of ethanol treatments, starting from the 70% storing solution, and then were cleared in xylene. The blocks were serially sectioned at 7 µm with a rotary microtome (Motorized rotary microtome, United States). The sections were stained with Hematoxylin-Eosin for general morphology assessment. The sum of Langerhans islands was obtained through counting the total island appeared in three microscopic fields under ×4 magnification.

### Statistical analysis

All values are displayed as mean ± S.E.M. Statistical analysis was accessed using OpenEpi (https://www.openepi.com/Menu/OE_Menu.htm). The results were compared using a one-way analysis of variance (ANOVA), and significant differences among means were tested using Student’s t-test. Only p-values less than 0.05 were considered statistically significant.

## Results

### Weight gain

**FIGURE 1:**
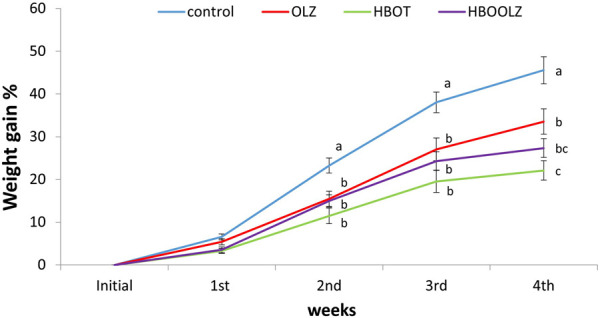
The cumulative percentage in weight gain in response to olanzapine intraperitoneal administration to SD female rats at a dose of 10 mg/kg/day in the presence and absence of HBOT. The values are means for the cumulative weight percentage ±S.E. The different letters indicate statistically significant differences between experimental groups at each time point (*p* < 0.05) (means shared the same letters were not significantly different).

Olanzapine treatment showed significant weight gain decrease below the control group that didn’t receive any therapy (i.e., HBOT nor OLZ) (Figure 1). The weight gain increment in control rats was 42.5% after 5 weeks of the beginning of the experiment. None of the other experimental groups that received OLZ treatment or the control rats that were placed on HBOT reached this percentage of weight gain. It was very interesting to mention that the effect of high level of oxygen exposure inside HBO chamber further hampered the growth percentage curve (weight gain) of the treated rats to almost half of the weight gain obtained in control animals by the end of the experiment. Interestingly, OLZ treated groups (i.e., with or without HBOT) caused a significant decrease in weight gain started at second weeks after treatment till the end of the experiment at 5 weeks.

### Fasting blood sugar (FBS), insulin (INS) level and insulin resistance or sensitivity indices

**FIGURE 2:**
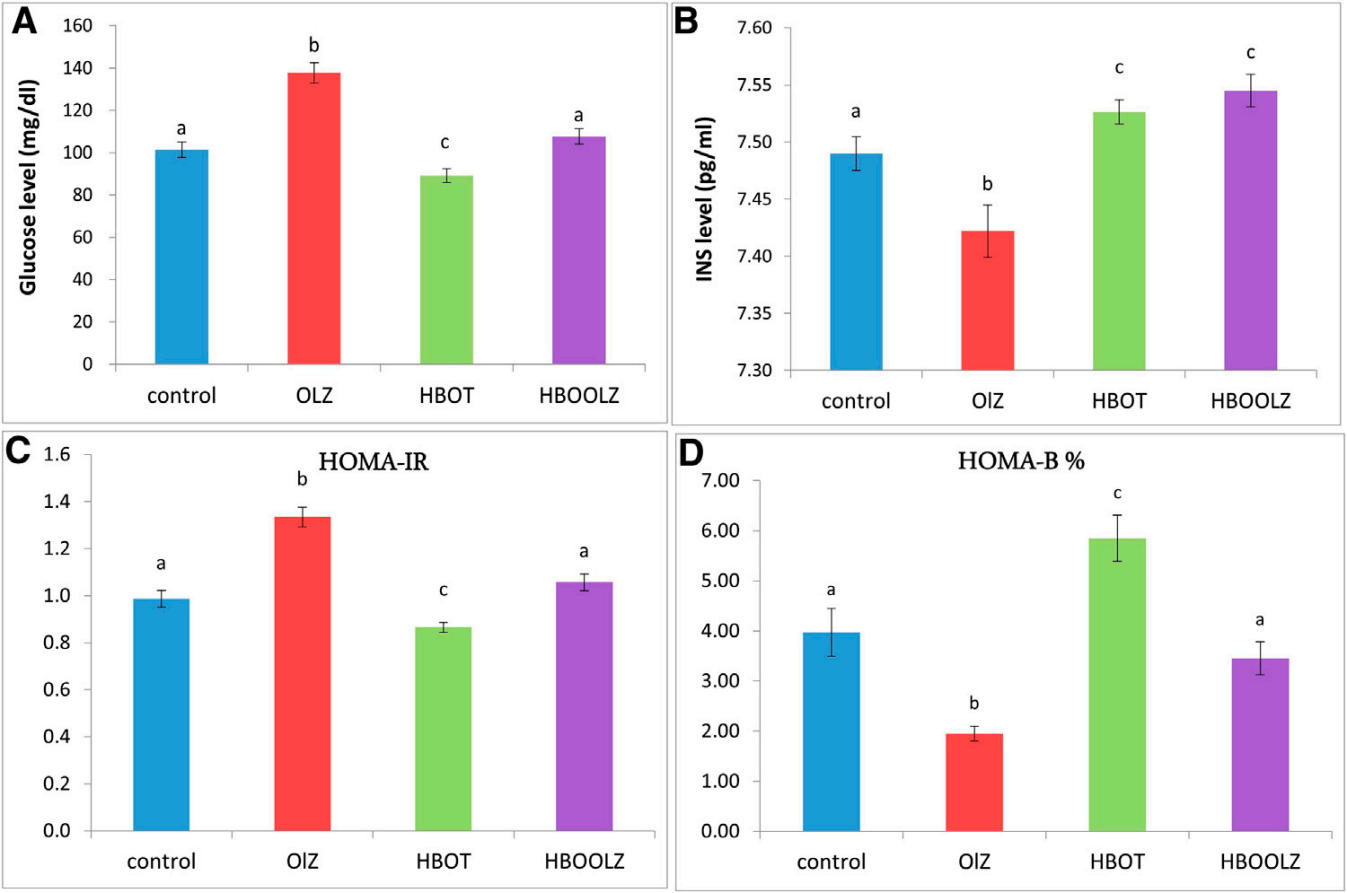
Effect of a daily single intraperitoneal dose of olanzapine (10 mg/Kg) for 5 weeks on serum fasting blood sugar (FBS) **(A)**, insulin (INS) level **(B)**. Insulin resistance markers in rats (HOMA-IR) **(C)** and insulin sensitivity markers (HOMA-B%) **(D)** for rats placed at different oxygen concentrations inside HBO. The values are means ± S.E. Different letters represent statistically significant differences (*p* < 0.05) (means shared the same letters were not significantly different).

The measurement of serum glucose level after fasting around 10 h (FBS) showed that there is an increment in animals that were injected by olanzapine compared with control group. The most interesting observation is that HBOT significantly lowered the glucose level in the OLZ treated group to the normal level. As well, HBOT to rats without OLZ treatment was the lowest among all experimental and control groups. On the other hand, insulin concentration in serum showed a minute difference among the groups (0.1 pg/ml). However, it was noticed that HBOT for rats caused a significant increase in insulin concentration regardless of the OLZ treatment, while OLZ treatment alone showed the lowest insulin concentration among all experimental groups (Figure 2). In regard to insulin resistance marker, rats on OLZ alone clearly had 20% higher HOMA-IR than control rats but when OLZ treatment is combined with HBOT (HBOOLZ) the HOMA-IR index return to normal levels of the control. Although serum insulin levels were comparable in all groups with slight significant decrease in OLZ group, it seems that the treatment effect get clearer when using HOMA-B index for comparison. This index was the lowest in the OLZ treated group and when HBOT is applied along with OLZ treatment the Index return to normal values indicated in control group.

### Lipid profile such as cholesterol, triglyceride (TG), LDL and HDL

**FIGURE 3:**
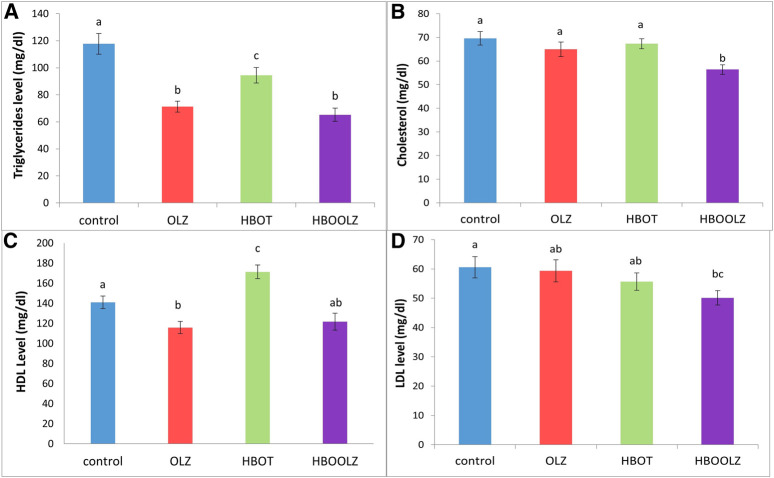
The effects of daily olanzapine intraperitoneal injection for 5 weeks on serum triglyceride (TG) **(A)**, cholesterol **(B)**, high density lipoprotein (HDL) **(C)** and low-density lipoprotein (LDL) **(D)** in the presence of different oxygen concentrations. The values are means ± S.E. Different letters represent statistical significant differences with a *p*-value less than 0.05.

The effect of oxygen therapy and injection of olanzapine on triglyceride (TG) concentration in rat serum showed a significant down-regulation of TG in these groups compared with control group. It is important to note that this decrease was most prominent when animals were received OLZ and regardless of HBOT (Figure 3A). The combination of OLZ treatment and HBOT causes slight decrease in serum cholesterol and LDL levels in comparison to other groups (Figures 3B, D). HDL concertation positively affected by HBOT alone while OLZ treatment negatively affected its concentration regardless of the HBOT (Figure 3C). It is also very interesting to mention that the effect of OLZ injection along with HBOT (HBOOLZ) caused significant decrease in LDL when only compared to control group (Figure 3D).

### Enzymatic tests

**FIGURE 4:**
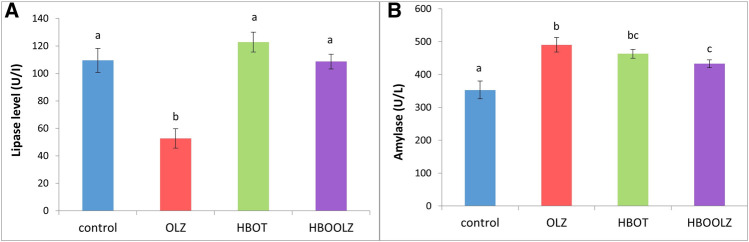
The effects of daily olanzapine intraperitoneal injection and HBOT for 5 weeks on rat’s serum pancreatic enzymes; Lipase enzyme **(A)** and Amylase enzyme **(B)**. The values are means ±S.E. Different letters represent statistically significant differences (*p* < 0.05) (means shared the same letters were not significantly different).

Olanzapine treatment caused significant decrease in serum lipase level, while exposure of these rats to high level of oxygen in HBO chamber can reverse this negative effect of OLZ (Figure 4A). All rats received OLZ treatment and/or HBOT showed a significant rise in serum amylase when compared with control group. Rats’ exposure to HBOT before OLZ injection (HBLOOZ group) result in decrease in amylase concentration below the level detected in OLZ treated group (Figure 4B) but still higher that the level detected in the control group.

## Histological changes of Langerhans cells in pancreas in response of OLZ and HBOT

**FIGURE 5:**
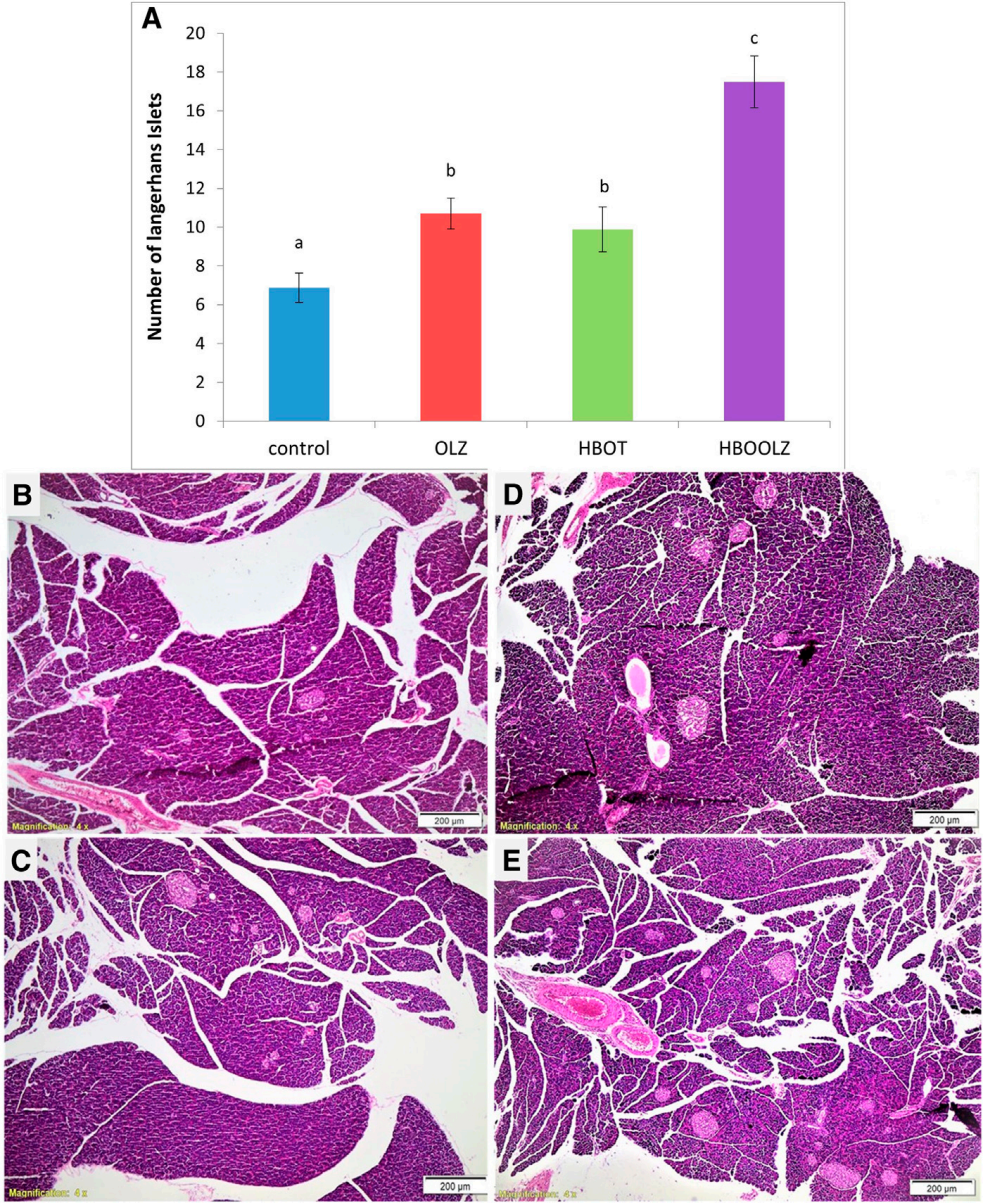
The effects of daily olanzapine intraperitoneal injection and HBOT for 5 weeks on number of Langerhans cells in pancreas **(A)**. The values are means of the total number of islands counted in three fields at ×4 magnification for the experimental animals in each group ±S.E. Different letters represent statistically significant differences (*p* < 0.05) (means shared the same letters were not significantly different). Histopathological changes in Langerhans islets of pancreas due to different interventions; control rats received neither OLZ nor HBOT **(B)**; rats injected with only OLZ **(C)**; HBOT group only received the oxygen therapy without OLZ treatment **(D)**; HBOOLZ received both OLZ and HBOT group **(E)**.

The effect of oxygen therapy and injection of olanzapine on number of Langerhans cells in pancreas showed a significant up-regulation in these groups compared with control group. It is important to note that the combination of OLZ treatment and HBOT almost doubled the Langerhans cells compared to normal tissue in control group (Figure 5).

## Discussion

This is the first study that examined the effects of hyperbaric oxygen treatment (HBOT) on olanzapine (OLZ)-induced metabolic disorders in rat model. This work also contains some novel findings that HBOT attenuates OLZ-induced blood biological side effects. The main findings are that olanzapine treated rats actually lose weight, but continue to exhibit metabolic dysregulation which appear to be alleviated by hyperbaric oxygen treatment. The oxygen treatment also appears to result in Langerhans islet proliferation in response to different treatments’ combinations. The main side effects of OLZ that got affected by HBOT were related to metabolic disorders including lipid profile, glucose and insulin levels, weight gain as well as some enzymatic changes related to metabolism such as lipase and amylase.

OLZ treatment lead to a decreased insulin sensitivity which mainly related to β-cell dysfunction [38]. It seems that OLZ treatment upregulated the number of cells in pancreatic tissues (Langerhans islets) but made them less functional in secreting insulin as presented by low HOMA-B value. This particular negative effect not only got reversed after HBOT application but also led in tremendous increase in Langerhans islets number in HBOOLZ group. To the best of our knowledge, this is also the first study the showed a direct dysfunctional effect of OLZ on pancreatic tissues that can be alleviated by HBOT application.

It was demonstrated that subjects placed on OLZ have an increase appetite that led later to an increment in weight gain. Patients that regulate their food intake (behavioral or by other medications) appetite usually had no weight difference after OLZ therapy [39]. In animal models, OLZ effect on animals is related to gender and is dose dependent [13, 42, 43]. Thus, the weight gain after OLZ treatment is controlled through behavioral changes that can be implemented when treated subjects control their food intake or can be manipulated in animal model by changing the administrated dose, interval, and route of administration. In the current study, animals were administrated OLZ therapy that adopted a high dose of OLZ (10 mg/Kg, daily for 35 days). This high dose led to decrease in rats’ activity and willingness to move toward feeders (observational). This locomotor inhibition was noticed in all treated animals (Data not shown). Similarly, it was well documented that human patients treated with OLZ lack the tendency to perform normal daily activities. The patients who suffer from schizophrenia for instance and consuming OLZ had a lower physical activity, poor fitness, muscular strength and sedentary lifestyle in addition to an altered body balance [14, 32, 33]. Despite the unwillingness for activities in human treated with OLZ, treated patients usually with increase appetite can be easily served the required meals from family or healthcare takers. This option is not available in animal models which might explain why in many animal models treated with OLZ, some animals with low activities lose weight or had no weight difference when compared to control groups [10, 11]. Consequently, as reported in current study, rats lost weight significantly starting from the third week of treatment till the end of the experiment (Figure 1).

HBOT and OLZ treated rats had same weight gain profile until week four of the experiment which later became significantly even lower than the OLZ treated group (Figure 1). It was expected that HBOT can induce some weight loss in animals placed on this therapy [44]. In fact, the use of HBOT in the current study was first used in order to prove this weight loss benefit in OLZ treated animals so it can be later adopted as a therapy in patients suffering from weight gain problem after OLZ treatment. Unfortunately, the OLZ treatment program that used high dose in this study could not mimic the same picture present in human subjects in regard to weight gain. However, the dose and the route of administration (intraperitoneal) applied in the current study considered the highest dose in rat model that can give the most side effects associated with OLZ treatments in rats [13] but it seems that it particularly did not induce weight gain in rats because of the decrease activity and subsequent decrease in food intake.

In relation to the previous findings, serum lipids were not elevated in the OLZ treated rats as expected in human subjects but instead was similar to the control group or even decreased compared specifically TG (Figure 3). This proves that the lipid profile disturbance associated with OLZ treatment is a direct effect of increase body weight due to increase in food consumption, which did not occur in this model. As mentioned in our methodology, this experiment is intended to study the changes happening while using high dose OLZ. High doses are well known to reduce movement and reduce food intake, which usually ends by internal consumption of fat [39]. HBOT effect on serum lipids was the most striking in the current study. The previous work showed that HBOT was useful in decreasing several metabolic disorders associated with increase lipid consumption in rat weight gain model [44]. Some parameters as cholesterol and LDL were still clearly decreased when OLZ treatment is associated with HBOT therapy. Therefore, the HBOT can be considered as a therapeutic approach to control hyperlipidemia problem associated with OLZ treatment in human subject. Further studies still need to proof this effect when the OLZ treatment when given at low dose to induce other metabolic disorders such as weight gain and hyperlipidemia.

The main findings in the current work are that olanzapine treated rats actually lose weight, but continue to exhibit metabolic dysregulation (fasting glucose and insulin levels) which appear to be mitigated by hyperbaric oxygen treatment. A study stated that OLZ is associated with high blood sugar levels which may be reversible after discontinuation of treatment [21, 45, 46]. It has been hypothesized that OLZ can decrease the responsiveness of the pancreatic β-cells or even induce pancreatic cell apoptosis in animal models or *in vitro* [11, 22, 47]. However, most human studies did not relate hyperglycemia associated with OLZ treatment with altered pancreatic β-cell histopathological changes *in vivo* [48]. It is generally considered that the development of diabetes after OLZ occur due to inducing obesity and subsequent insulin resistance [12, 18, 19]. However, in some patients treated with OLZ, hyperglycemic developed without reporting weight gain which is similar to certain extent to the data reported in the current study [49, 50].

It was revealed that the serum fasting blood sugar (FBS) significantly increased in the rats that treated with OLZ, while applying HBO therapy effectively alleviated such changes. Hereby, the groups of rats which were treated in the hyperbaric oxygen (HBO) chamber alone showed a significant decrease in the level of FBS slightly below the level detected in control rats, while rats which were placed in HBO chamber then injected with OLZ showed that the FBS level returned to the normal level. This outcome might be due to increase in the level of insulin in serum when exposing animals to high level of oxygen under pressure in HBO chamber. It was also noticed that HBOT to rats caused a significant increase in insulin concentration above control levels regardless of the OLZ treatment. The OLZ treatment alone showed the lowest insulin concentration among all experimental groups. It is known that a high level of insulin is accompanied by a decrease in the level of glucose in the serum. This is what was shown in a previous study in which patients were given HBOT for 2 h, six sessions per week for 5 weeks had an increase in insulin sensitivity within 3 days of hyperbaric oxygen treatment [51]. Similarly, HBOT was able to improve insulin sensitivity and reverse insulin resistance along with many other MDs parameters rats with abdominal obesity, induced by sucrose induced metabolic syndrome in rats [52].

The insulin level after OLZ treatment alone in the current work was slightly down regulated but it still within the normal physiological level in rats. This normal insulin level did not though result in controlling the significantly upregulated serum glucose level in OLZ treated group which is translated by the increased HOMA-IR index in these animals. Particularly, blockage of various receptors by olanzapine involves the regulation of glucose metabolism independent of their effects on body weight. Olanzapine blocks serotonin 5-HT2, histamine H1, α-1 adrenergic, D2 dopamine receptor (D2R) and muscarinic M3 receptors (M3R) [53]. These receptors regulates glucose uptake by muscle tissues (e.g., H1) [54], glucose tolerance (e.g., α-1 adrenergic) [55], glucose-stimulated insulin secretion by pancreatic β-cells and tissue insulin resistance and diabetes (D2R and M3R) [56–60] which clearly reflected by low HOMA-B index in OLZ treated rats in the current study. The findings reported in the current study presented insulin-resistance effect of OLZ while application of HBOT were able to alleviate these effect probably through activation of various receptors mentioned above which all need further investigations.

In the current study, the Langerhans Islet number was proliferated in pancreatic tissues after treatment with OLZ and HBOT (Figure 5). To the best of our knowledge, no *in vivo* study on OLZ showed such effect on Langerhans Islet. Thus, the increase in number of Langerhans Islet in pancreatic tissues of these rats may suggest that the pancreas is probably going into hyperplasia to compensate for the insulin resistance which is usually associated with OLZ treatment. The islet of Langerhans usually undergoes major structural and functional changes to control glucose level. It was reported that after HBOT therapy many adaptive changes that results in enhancement of glucose stimulated insulin secretion, β-cell proliferation, glucose oxidation and cAMP metabolism. It seems that HBOT had a significant effect on the number of islets that result in normalizing glucose concentrations and put insulin sensitivity back to normal levels. The present study provides evidence that HBOT therapy mitigated the metabolic side-effects related to OLZ treatment and specifically seems to control insulin resistance probably through proliferation of the Langerhans Islet in pancreatic tissues and increase their activities.

The type of cells that got proliferated need further analysis, as the main two types of cells present in the islets, β-cells and α-cells have opposite effects. The current data in two different treatment groups (HBOT and OLZ) gave similar effect on islets proliferation but resulted in different outcome on glucose and insulin production. Therefore, close analysis of islets of Langerhans should be examined in further studies along with the investigation of whether HBOT can reverse the reported antagonistic effect of OLZ on various receptors involve in the regulation of glucose metabolism and uptake.

In the present study, the activity of pancreatic enzymes in rats’ serum was affected by the OLZ treatment, the amylase activity increased while lipase enzyme activity decreased. The reduction of serum lipase is associated with decrease TG in animals administered OLZ. In other words, less feed intake means less demand on pancreas to release lipase. This is clearly related with the low TG presence in serum after OLZ treatment reported in this study. However, it was clearly demonstrated that up-regulation in serum amylase is related to the presence of acute Pancreatitis [61]. Therefore, the decrease in amylase production when OLZ treatment is combined with HBOT indicates that this therapy can control the acute pancreatitis associated with OLZ treatment [25, 62]. As well, our results are consistent with other studies that found HBOT had beneficial effects on inflammatory disease such as acute pancreatitis [63–65]. Therefore, close analysis for inflammatory process in the pancreas is worth later investigation.

A study recommend that people who take olanzapine, especially for long periods of time, should exercise to improve their clinical symptoms, quality of life and depressive symptoms [66] Unfortunately, most subjects could not regularly exercise due to poor motivation as the main reason or muscle weakness and tardive dyskinesia (uncontrolled body movements) which occur during or after treatment with olanzapine. On the other hand, the current results showed consistent findings with other studies that found HBOT had beneficial effects on the biochemical and histological abnormalities related to metabolic disorders and even can improve several pathological processes that are relevant to acute pancreatitis. Therefore, we can recommend adoption of HBOT before OLZ administration to improve metabolic dysregulation and others side effect that associated with OLZ treatment. As this study was novel in introducing such protocol in animal model, further studies need to replicate this research in human patients to adopt a proper protocol in human. The HBO individual chambers can be then owned by patients and the therapy can be adapted and applied at home as daily routine protocol to alleviate patients undesired side effects associated with OLZ treatment and as an alternative to indoors exercising devices.

## Data Availability

The raw data supporting the conclusion of this article will be made available by the authors, without undue reservation.
